# The status of invasive pneumococcal disease among children younger than 5 years of age in north-west Lombardy, Italy

**DOI:** 10.1186/1471-2334-12-106

**Published:** 2012-05-03

**Authors:** Enrica Riva, Filippo Salvini, Maria Laura Garlaschi, Giovanni Radaelli, Marcello Giovannini

**Affiliations:** 1 Department of Pediatrics, San Paolo Hospital, University of Milan, Via A. Di Rudinì 8, I-20142, Milan, Italy; 2 Microbiology Laboratory, Fondazione IRCCS Ospedale Maggiore Policlinico Mangiagalli e Regina Elena, Via della Commenda 10, I-20122, Milan, Italy; 3 Unit of Medical Statistics, San Paolo Hospital, University of Milan, Via A. Di Rudinì 8, I-20142, Milan, Italy

## Abstract

**Background:**

*Streptococcus pneumoniae* is a leading cause of invasive infection in young children causing morbidity and mortality. Active surveillance systems of invasive pneumococcal disease (IPD) are recommended worldwide. The aim of this study was to estimate the current incidence of IPD and to describe the serotype distribution and the antimocrobial susceptibility of *S. pneumoniae* isolates in children aged less than 5 years residing in North-West Lombardy, Italy.

**Methods:**

A twelve-month prospective active surveillance system recruited all children aged less than 5 years admitted for suspicion of IPD at emergency room of ten hospitals located in the monitored area. Blood samples were taken in all participants for confirmation of IPD based on isolation of *S. pneumoniae* from blood. Pneumococcal meningitis and sepsis were additionally confirmed by cerebrospinal fluid analysis. Serotyping and antimicrobial susceptibility testing were performed on isolates from blood.

**Results:**

A total of 15 confirmed cases of IPD were detected among 135 recruited children, including pneumonia (n = 8), bacteremia (n = 4), sepsis (n = 2) and meningitis (n = 1). The annual IPD incidence rate was 50.0/100,000 (95%CI, 30.5-82.5/100,000). Incidence was 58.3/100,000 (28.8-120.1/100,000) among children aged less than 2 years and 44.4/100,000 (22.9-87.5/100,000) among children aged 2–4 years. Thirteen isolates were typified. The most common serotype was 19A (23.1%) that together with serotypes 1, 7F and 19F accounted for 69.2% of typified isolates. Serotypes 14, 23F, 12B and 15C were also identified. The 7- and 13-valent pneumococcal conjugate vaccines covered respectively 30.8% and 84.6% of typified IPD cases. One isolate (serotype 15C) was penicillin-resistant and caused meningitis.

**Conclusions:**

The inclusion of the 13-valent pneumococcal conjugate vaccine in immunization programs of young children might be considered to reduce incidence and morbidity of invasive pneumococcal disease in this surveilled population.

## Background

*Streptococcus pneumoniae* (*S. pneumoniae)* is a leading cause of bacterial pneumonia, sepsis and meningitis in children, and is associated with high morbidity and mortality. Recent estimates of deaths caused by *S. pneumoniae* in children younger than 5 years range from 700,000 to 1 million every year worldwide [1-3], with a fatality rate of around 11% (excluding pneumococcal deaths in human immunodeficiency virus positive children) [4].

Decline in the number of cases of invasive pneumococcal disease (IPD) has been observed among children, especially in infants, both in USA and European countries which introduced the hepta-valent pneumococcal conjugate vaccine (PCV) in their immunization programs [5-8], with higher reduction in USA, where also reduction in IPD mortality occurred [9]. Contemporarily, an increase in the frequency of serotypes not included in PCV7 has been observed [7,10], above all of serotype 19A [11-15]. Additionally, the circulating serotypes vary across geographical areas and may dynamically evolve, resulting in different vaccine coverage [2,16,17]. Therefore, as pneumococcal vaccines provide protection in a serotype-specific manner, their appliance should be based also on the knowledge of actual circulating isolates [14]. Prospective studies would be desirable to hopefully help the health Authorities in planning efficient immunization strategies, and the industry to possibly arrange new updated vaccines. Indeed, the World Health Organization recommends currently countries to conduct appropriate surveillance of IPD to estimate the vaccine coverage rate and to monitor continuously the effect of vaccination [2]. In Italy, few local prospective studies have been conducted in children aged less than 5 years [18-21]. There is lack of longitudinal data in Lombardy, a crucial region with around 9,000,000 resident people.

The main objectives of this study were to estimate the current incidence of IPD in children aged less than 5 years in North-West Lombardy, Italy, and to describe the serotype distribution of *S. pneumoniae* isolates and antimicrobial susceptibility. These data will allow to guide use of different pneumococcal conjugate vaccines in young children in this region.

## Methods

### Subjects

This prospective, multicenter, observational study was conducted throughout the first 12-month period of an ongoing active surveillance system of IPD in young children in North-West Lombardy, including the city of Milan, Italy, and started on September 1, 2008. The study involved 10 hospitals representatively distributed in the territorial area delimited by four Reference Local Health Authorities districts serving at the beginning of the study around 3,500,000 people and comprising 130,000 children aged less than 5 years of whom 30,000 (12,000 younger than 2 years of age) linked to the participant hospitals. All children admitted at emergency room of hospitals were visited carefully and assessed for eligibility. Inclusion criteria were: age at recruitment less than 5 years; being residing in the monitored area; reporting suspicion of IPD, namely, any *S. pneumoniae* clinical syndrome (e.g., pneumonia, bacteremia, sepsis, or meningitis), and/or (in children aged ≤36 months) having at admission a measured rectal temperature or history of a measured temperature >39.0°C within 24 hours before; negative urinalysis for urinary tract infections based on fast urine test; C-reactive protein >15 mg/L. The exclusion criterion was refusal to give written consent. The complementary inclusion criterion and/or temperature >39°C was considered in children aged ≤ 36 months as this condition may be *per se* suggestive of IPD within this range of age, independently of other clinical signs or symptoms [22].

Urinalysis was performed by Aution sticks-10EA (ARKRAY Europe, B.V., Amstelveen, The Netherlands). C-reactive protein was measured by direct immunoturbidimetry (Tina-Quant, Roche Diagnostics, Hoffmann-La Roche Ltd, Basel, Switzerland).

The study was conducted in accordance with the Declaration of Helsinki and Good Clinical Practice guidelines. The ethics committee of each hospital reviewed and approved the study protocol. Written informed consent was obtained at recruitment from the parents or legal guardians. No incentive was provided to encourage study participation.

### Baseline data

An electronic-case report form was used to record baseline information, including socio-demographic characteristics of family, breastfeeding, co-morbidity conditions, exposure to smoke, attending a community, previous pneumococcal vaccination of child, and antibiotics administration within the week before admission.

### Case definition

A case of confirmed IPD was defined in accordance with the Centers for Disease Control and Prevention (Atlanta, GA, USA) as isolation of *S. pneumoniae* from a normally sterile body site, such as blood or cerebrospinal fluid (CSF) [23]. Diagnosis of specific invasive pneumococcal disease was in accordance with the International Classification of Diseases [ICD]-9 [24] that is pneumonia (481), bacteremia (790.7 and 041.2 *or* 038.9 and 041.2), sepsis (038.2), or meningitis (320.1 *or* 320.8 and 041.2). In particular, the definition for pneumococcal meningitis was isolation of *S. pneumoniae* from cerebrospinal fluid or the clinical diagnosis of meningitis with pneumococcus isolated from another normally sterile site [25].

### Identification of isolates

Blood samples were taken in all participant children within 24 h of recruitment. CSF samples were additionally taken in children with clinical syndrome of sepsis or meningitis. *S. pneumoniae* isolates were analyzed and identified by using standardized laboratory procedures, including colony morphology on blood agar, the optochin test and the sodium deoxycholate solubility test. Fresh cultures of isolates on blood agar plates and in Amies medium swab were sent to the Pneumococcal Reference Laboratory in Lombardy (Milan, Italy) for analysis. At the reference laboratory, all the isolates were confirmed as *S. pneumoniae* by testing for alpha haemolysis on blood agar, optochin susceptibility and bile solubility. Serogrouping was then performed on each isolate using a commercial kit for latex agglutination (Pneumotest latex kit, Statens Serum Institut, Copenhagen, Denmark). The agglutination kit contains latex particles coated with rabbit antibodies reacting with specific pneumococcal capsular polysaccharide and identification of pneumococcal serogroups follows a checkerboard system after agglutination in 14 pool suspensions.

### Serotyping of isolates

Omni serum (Statens Serum Institut) includes antibodies to all recognized *S. pneumoniae* serotypes. A suspension of the test organism was prepared in saline solution 0.9% from well isolated colonies grown on sheep blood agar plates for 18 to 24 h in 5% CO_2_ at 35°C. One drop of this suspension was mixed with 1 drop of antiserum and 1 drop of methylene blue and, after incubation at room temperature for 10 min, examined at 1,000x magnification. Visible evidence of capsular swelling with Omni serum, i.e., a positive Quellung reaction, was considered positive, and followed by type specific antisera.

### Antimicrobial susceptibility testing

Antimicrobial susceptibility testing of isolates against penicillin G, ceftriaxone, erythromycin, tetracycline, trimethoprim-sulfamethoxazole, chloramphenicol, levofloxacin, linezolid and vancomycin was performed using the E test method (AB Biodisk, Solana, Sweden) on Mueller-Hinton agar supplemented with 5% defibrinated sheep blood (bioMérieux Italia Spa, Bagno a Ripoli, Italy). Inocula were prepared by suspending pneumococcal colonies to a density that matched a 0.5 McFarland opacity in Trypticase Soy Broth (bioMérieux Italia Spa). The minimal inhibitory concentration was tested following the manufacturer’s instructions and interpreted in accordance with the Clinical and Laboratory Standards Institute (CLSI) breakpoints [26]. *S. pneumoniae* ATCC 49619 was used as the quality control strain in each run, as recommended by CLSI [26].

### Statistical analysis

Comparison between confirmed and non-confirmed IPD groups was performed by the Fisher’s exact test or the nonparametric Mann–Whitney *U* test, as appropriate. The incidence rate of IPD was estimated by the number of laboratory confirmed IPD cases divided the number of children monitored in the sampled population at the beginning of the study. Exact binomial 95% confidence intervals (CI) were calculated. A *P-*value less than 0.05 was considered to indicate statistical significance (two-tailed test). The statistical analyses were done using the SPSS software, version 17.0 (SPSS Inc., Chicago, IL).

## Results

All parents or legal guardians of children gave written consent to participate in the study. A total of 135 children was recruited (75 boys, 60 girls; mean age [SD ] 2.5 [1.4] years). Blood testing data were available in 133 (98.5%) cases, and 15 were positive for *S. pneumoniae*, i.e., 11.3% (95%CI, 6.9-17.8%). There were 7 cases of confirmed IPD in children aged less than two years (infants) (12.3%) and 8 cases in children aged 2–4 years (12.5%). All of them were hospitalized. No fatality outcome occurred. The overall annual incidence of confirmed IPD was 50.0/100,000 (95%CI, 30.5-82.5/100,000). Incidence was 58.3/100,000 (28.8-120.1/100,000) and 44.4/100,000 (22.9-87.5/100,000) in infants and children aged 2–4 years, respectively.

Table 1 shows the baseline characteristics of recruited children by IPD status. The overall rate of previous pneumococcal vaccination was 34.8% (95%CI, 27.3-43.2%) without significant difference between children with confirmed or non-confirmed IPD. Co-morbidity conditions were more frequently observed in children with confirmed IPD. No child was human immunodeficiency virus positive.

**Table 1:** Baseline characteristics of the recruited children by IPD status

**Characteristic**		**IPD†**		
	**All recruited (n = 135)**	**Non-confirmed (n = 118)**	**Confirmed (n = 15)**	***P*-value**^**§**^
Age (y)				0.787
<2	58 (43.0)	50 (42.4)	7 (46.7)	
2-4	77 (57.0)	68 (57.6)	8 (53.3)	
Sex (boy)	75 (55.6)	63 (53.4)	11 (73.3)	0.175
Race (White)^§§^	116 (85.9)	101 (85.6)	13 (86.7)	1.000
Having been breastfed (yes)	103 (76.3)	92 (78.0)	10 (66.7)	0.340
Siblings (yes)	68 (50.4)	62 (52.5)	5 (33.3)	0.182
Having a parent smoking (yes)	31 (23.0)	29 (24.6)	2 (13.3)	0.519
Attending a community (yes)	54 (40.0)	45 (38.1)	8 (53.3)	0.276
Co-morbidity conditions^§§§^ (yes)	2 (1.5)	---	2 (13.3)	0.012*
Having received pneumococcal vaccine (yes)	47 (34.8)	41 (34.7)	4 (26.7)	0.773
Type (conjugate/polysaccharide)	41/6	36/5	3/1	0.448
Doses (n)^§§§§^				0.551
1	15 (31.9)	14 (34.1)	1 (25.0)	
2	18 (38.3)	17 (41.5)	1 (25.0)	
≥3	14 (29.8)	10 (24.4)	2 (50.0)	
Median (range) age at the first dose of vaccine (mo) ^§§§§^	6.5 (2–40)	6.0 (2–35)	9.5 (4–40)	0.250
Having received antibiotic therapy during the week before recruitment (yes)	40 (29.6)	38 (32.2)	1 (6.7)	0.067

Data are number (percentage within group) of children, except age at the first vaccination.

†In two cases the IPD status was not available.

§Significance of difference between confirmed and non-confirmed IPD groups (Fisher’s exact test or Mann Whitney *U* test). *: Statistically significant.

^§§^Other races included Afro/Afro-American (n = 13), Asian (n = 1) and mixed-races (n = 5).

^§§§^Sickle cell anaemia (n = 1) and cardiovascular disease. Both children did not receive pneumococcal vaccine.

^§§§§^ In children who received pneumococcal vaccine.

All the 15 confirmed IPD cases had *S. pneumoniae* isolated from blood; 53.3% exhibited pneumonia, 26.7% bacteremia, 13.3% sepsis and 6.7% meningitis. Both meningitis and sepsis were as well confirmed by CFS analysis. No significant difference was found between infants and children aged 2–4 years with respect to diagnosis or clinical signs of infection (Table 2).

**Table 2:** Clinical diagnosis and characteristics of infection in confirmed IPD cases, by age group

		**Age (y)**		
	**Confirmed IPD**	**<2**	**2-4**	
	**(n = 15)**	**(n = 7)**	**(n = 8)**	***P*-value**^**§**^
Diagnosis (ICD-9)				0.189
Pneumonia	8 (53.3)	2 (28.6)	6 (75.0)	
Bacteremia	4 (26.7)	2 (28.6)	2 (25.0)	
Septicemia	2 (13.3)	2 (28.6)	---	
Meningitis	1 (6.7)	1 (14.2)	---	
Rectal body temperature >38°C (yes)	13 (86.7)	6 (85.7)	7 (87.5)	1.000
C-reactive protein (mg/L)				1.000
10–40	3 (20.0)	1 (14.2)	2 (25.0)	
41–200	7 (46.7)	4 (57.2)	3 (37.5)	
>200	5 (33.3)	2 (28.6)	3 (37.5)	
Median (range) leucocytes (x10^9^/L)	22.4 (12.9-41.0)	29.2 (17.8-41.0)	20.7 (12.9-26.4)	0.189

Data are number (percentage within group) of children, except leucocytes.

§Significance of difference between age groups (Fisher’s exact test or Mann Whitney *U* test).

Thirteen (86.7%) isolates were typified while 2 cases were not available for serotyping. The serotype distribution in typified isolates and susceptibility against antibiotics are reported in Table 3. The most observed serotype was 19A, that accounted 23.1% of typified isolates. In infants, all detected serotypes were found except serotypes 1 and 19F. In children aged 2–4 years serotypes 19A, 1, 7F, and 19F accounted 100% of typified isolates. Serotypes 1, 14, 7F and 19F caused pneumonia, serotypes 19A and 19F bacteremia, and serotypes 12B and 23F sepsis. The serotype 15C caused meningitis and showed antimicrobial resistance to penicillin and tetracycline. Four children with serotyped isolates (serotype 19A, n = 2; serotype 1, n = 2) had received pneumococcal vaccine previously (conjugate vaccine, n = 3; polysaccharide vaccine, n = 1). In five children, who never had received any pneumococcal vaccine (age < 2 years, n = 3; age 2–4 years, n = 2), the serotype showed any antibiotic resistance (Table 3).

**Table 3:** **Serotype distribution of*S. pneumoniae*typified isolates by age group, and antimicrobial susceptibility by antibiotic**

		**Age group (y)**		**Resistant isolates according to CLSI criteria†**		
**Serotype**	**Confirmed IPD**	**<2**	**2-4**	**Penicillin**	**Erytromicin**	**Tetracycline**
	**(n = 13)**	**(n = 7)**	**(n = 6)**			
19A	3 (23.1) [2]	2 (28.6) [1]	1 (16.7) [1]	---	1 (7.7)	---
1	2 (15.4) [2]	---	2 (33.3) [1]	---	---	---
7F	2 (15.4)	1 (14.3)	1 (16.7)	---	---	---
19F	2 (15.4)	---	2 (33.3)	---	2 (15.4)	2 (15.4)
14	1 (7.7)	1 (14.3)	---	---	1 (7.7)	---
23F	1 (7.7)	1 (14.3)	---	---	---	---
12B	1 (7.7)	1 (14.3)	---	---	---	---
15C	1 (7.7)	1 (14.3)	---	1 (7.7)	---	1 (7.7)
Total	13 (100)	7 (100)	6 (100)	1 (7.7)	4 (30.8)	3 (23.1)

Data are number (column percentage) of cases. Number of cases having received any pneumococcal vaccine previously within square brackets

**†**Clinical and Laboratory Standards Institute [26.] The overall coverage rate, estimated on typified IPD cases, was 30.8% for PCV7 and 84.6% for PCV13. The coverage rate of PCV7 and PCV13 was, respectively, 28.6% and 71.4% in infants, and 33.3% and 100% in children aged 2–4 years.

The overall coverage rate, estimated on typified IPD cases, was 30.8% for PCV7 and 84.6% for PCV13. The coverage rate of PCV7 and PCV13 was, respectively, 28.6% and 71.4% in infants, and 33.3% and 100% in children aged 2–4 years.

## Discussion

Incidence of IPD in young children vary widely worldwide [2-4]. Decline has been observed in countries which introduced immunization programs based on the PCV7 [5-8]. Continuous surveillance of *S. pneumoniae* isolates causing invasive disease is necessary to evaluate the actual effect of vaccine [2] and to identify the circulating serotypes. This is the first active surveillance study conducted in Lombardy, Italy, to estimate the incidence of IPD in children aged less than 5 years and to determine the serotype distribution of *S. pneumoniae* isolates. In the monitored area the estimated annual incidence of IPD in children aged less than 5 years was 50.0/100,000. It was around 31% higher in infants than children aged 2–4 years.^.^ In Italy, few prospective regional studies have been conducted [18-21]. In children aged less than 5 years, incidence of IPD varied from 2.8/100,000 in Puglia (South Italy) to 5.7/100,000 in Piemonte [18]. In children aged ≤36 months the incidence was 58.9/100,000 in North-East [20]. The incidence of IPD in young children reported from different European countries before the introduction of pneumococcal vaccination ranged from 3.1/100,000 in Portugal to 110.2/100,000 in Spain (pooled 31.1/100,000) [27], with higher values in infants. Rates of IPD were lower in Europe than USA, where blood culture is performed for every febrile young child. In 1998–1999, before of introduction of the PCV7, the IPD incidence in children aged less than 5 years was 96.7/100,000. After the introduction of PCV7, incidence declined to 23.9/100,000 in 2003, with a percentage reduction of 75% [28]. Although in Europe there is lack of extensive epidemiological data on the effect of PCV7, a recent study estimated that an infant vaccination program would prevent 39,000 IPD cases in the 20 years after PCV7 introduction in the UK [29]. In Denmark, one year after introduction (2008) of PCV7, incidence of IPD declined of about 57% in infants and of 10% in children aged 2 years or older [8]. In the Netherlands a reduction of 44% was found two years after introduction of PCV7 in infants [7]. The PCV7 was introduced in Italy in 2001 and firstly recommended in at-risk children aged less than 5 years. Currently, either risk-based or universal vaccination programs are used, depending on the region, and PCV7 is offered either free of charge or with cost sharing [30]. Unfortunately, there is still paucity of information on the epidemiological effect of PCV7 [21].

Heterogeneity of IPD serotypes was observed in this study. Less than a third of the isolates belonged to serotypes covered by PCV7 while around 85% of them was covered by PCV13. In particular, the serotype 19A accounted 23.1% of typified IPD isolates. This result is comparable with recent national pooled data that estimated 19.2% of pneumococcal meningitis or sepsis occurred among children aged less than 5 years during in 2009 in Italy being caused by the serotype 19A [31]. A recent study conducted in Massachusetts showed throughout 2001–2007 period an increase in frequency of serotype 19A, that was the most cause of identified IPD cases in children (28%) [11]. The relevance of this emerging serotype, included in PCV13 but not in PCV7, has been as well indicated in South-West Asian countries [12,13], and emphasized recently [14,15,31,32].

The coverage of PCV7 was lower in this study (30.8%) compared to values reported from European countries. In Europe, PCV7 coverage in children aged less than 5 years ranges from 50% in Spain and Sweden to 100% in Greece, with a mean of 71%. In infants, the coverage of PCV7 ranges from 37% in Norway to 100% in Greece, with a mean of 72% [27]. The introduction of the PCV13 showed average increments of additional coverage of about 16% in children aged less than 2 years over PCV7 [2,27]. A study conducted in Spain in children aged less than 5 years showed that compared to PCV7 the PVC13 would increase the coverage by 17% (due to serotypes 1, 3, 5 and 7F) [33]. It may be handy to point out that in the population examined in this study, the 23-valent pneumococcal polysaccharide vaccine would show the same coverage of PCV13. However, it should be noted that current international guidelines recommend in children aged less than 5 years initial administration of the conjugate vaccine, above all in infants [25,34]. Indeed, infants may show poor antibody response to the polysaccharide vaccine [35] and level of antibody may decline already one year after polysaccharide vaccination [36].

The antimicrobial susceptibility of different serotypes of *S. pneumoniae* isolates may vary across geographic areas. In this study about 31% (4/13) of typified isolates was resistant to erythromycin (serotypes 14, 19A and 19F), 23% (3/13) was resistant to tetracycline (serotypes 19F and 15) and one isolate (serotype 15C) was resistant to penicillin G. In Europe the rate of penicillin G resistant serotypes in children aged less than 5 years ranges from 5% in Denmark to 55% in the Czech Republic (average 29%) [27]. Penicillin G resistance was often associated with the serotypes 19A (especially in Germany) and 14 in Poland [27]. For children aged less than 5 years, the mean proportion of erythromycin-resistant isolates was 35% (range 7% in Denmark to 53% in Spain), and erythromycin resistance was often associated with pneumococcal serotype 14 [27]. A warning may emerge from this study about the serotype 15C, as also reported in USA [37]. This serotype, currently not included in any available pneumococcal vaccine, caused meningitis and was resistant to penicillin and tetracycline.

Lastly, it is important highlight that all four IPD children, who had received pneumococcal vaccine previously, exhibited serotypes (19A and 1) covered by PCV13 but not by PCV7. On the whole, this information and the above results might be helpful in determining which conjugate vaccine is preferable for this population. Caution should be exercised anyway in inferring any definite conclusion due to the small number of isolates examined (n = 13). The small sample size of the current data base of IPD cases is a limitation, indeed. Continuous active surveillance, possibly based on a more branched network would be desirable to evaluate accurately the burden of disease caused by *S. pneumoniae* in this population, and to assess the effect of the vaccine on the incidence of IPD.

## Conclusions

This prospective active surveillance study on IPD conducted in Lombardy, Italy, in children aged less than 5 years proves the current burden of pneumococcal invasive disease in younger children and reveals the diversity of circulating *S. pneumoniae* serotypes, and may suggest possibly a potential additional beneficial effect of the PCV13 with respect to PCV7. The inclusion of the 13-valent pneumococcal conjugate vaccine in immunization programs might be considered to reduce the incidence and morbidity of invasive pneumococcal disease in this geographical area, in the younger population.

## Competing interests

This is a sponsored study funded by Pfizer Italia. GR is a Pfizer Inc. shareholder. All other authors declare they have no conflict of interest.

## Authors’ contributions

MG and ER made substantial contribution to the study conception and design, and were involved in drafting the manuscript. MLG planned the management and analyses of laboratory testing and was involved in drafting the manuscript. GR made substantial contributions to conception and design of the study, performed the statistical analysis, was involved in drafting the manuscript, and revised fully the final version of the manuscript. FS made substantial contribution to acquisition of data and was involved in drafting the manuscript. All authors gave final approval of the version to be published.

## Pre-publication history

The pre-publication history for this paper can be accessed here:

http://www.biomedcentral.com/1471-2334/12/106/prepub
